# Evaluation of Surgical Skills during Robotic Surgery by Deep Learning-Based Multiple Surgical Instrument Tracking in Training and Actual Operations

**DOI:** 10.3390/jcm9061964

**Published:** 2020-06-23

**Authors:** Dongheon Lee, Hyeong Won Yu, Hyungju Kwon, Hyoun-Joong Kong, Kyu Eun Lee, Hee Chan Kim

**Affiliations:** 1 Interdisciplinary Program, Bioengineering Major, Graduate School, Seoul National University, 101 Daehak-ro, Jongno-gu, Seoul 03080, Korea; dhlee@melab.snu.ac.kr; 2 Department of Surgery, Seoul National University Bundang Hospital, 82, Gumi-ro 173 Beon-gil, Bundang-gu, Seongnam-si, Gyeonggi-do 13620, Korea; hyeongwonyu@gmail.com; 3 Department of Surgery, Ewha Womans University Medical Center, 1071 Anyangcheon-ro, Yangcheon-Gu, Seoul 07985, Korea; lovekkung@gmail.com; 4 Department of Biomedical Engineering, Chungnam National University Hospital & College of Medicine, 282 Munhwa-ro, Jung-gu, Daejeon 301-721, Korea; 5 Institute of Medical & Biological Engineering, Medical Research Center, Seoul National University College of Medicine, 101 Daehak-ro, Jongno-gu, Seoul 03080, Korea; hckim@snu.ac.kr; 6 Department of Surgery, Seoul National University Hospital and College of Medicine, 101 Daehak-ro, Jongno-gu, Seoul 03080, Korea; 7 Department of Biomedical Engineering, Seoul National University College of Medicine, 101 Daehak-ro, Jongno-gu, Seoul 03080, Korea

**Keywords:** surgical skills, robotic surgery, deep learning, surgical instrument tracking, quantitative evaluation

## Abstract

As the number of robotic surgery procedures has increased, so has the importance of evaluating surgical skills in these techniques. It is difficult, however, to automatically and quantitatively evaluate surgical skills during robotic surgery, as these skills are primarily associated with the movement of surgical instruments. This study proposes a deep learning-based surgical instrument tracking algorithm to evaluate surgeons’ skills in performing procedures by robotic surgery. This method overcame two main drawbacks: occlusion and maintenance of the identity of the surgical instruments. In addition, surgical skill prediction models were developed using motion metrics calculated from the motion of the instruments. The tracking method was applied to 54 video segments and evaluated by root mean squared error (RMSE), area under the curve (AUC), and Pearson correlation analysis. The RMSE was 3.52 mm, the AUC of 1 mm, 2 mm, and 5 mm were 0.7, 0.78, and 0.86, respectively, and Pearson’s correlation coefficients were 0.9 on the *x*-axis and 0.87 on the *y*-axis. The surgical skill prediction models showed an accuracy of 83% with Objective Structured Assessment of Technical Skill (OSATS) and Global Evaluative Assessment of Robotic Surgery (GEARS). The proposed method was able to track instruments during robotic surgery, suggesting that the current method of surgical skill assessment by surgeons can be replaced by the proposed automatic and quantitative evaluation method.

## 1. Introduction

Most types of robotic surgery require training, with a classic learning curve eventually resulting in consistent performance [1]. It is important to repeatedly evaluate the surgical skills of each surgeon learning robotic surgical procedures to determine that surgeon’s current position on the learning curve. Surgical proficiency in laparoscopic surgery, a type of minimally invasive surgery, has been evaluated using the Objective Structured Assessment of Technical Skill (OSATS) [2]. Moreover, proficiency in robotic surgery, currently the primary type of micro-invasive surgery, has been evaluated using the Global Evaluative Assessment of Robotic Skills (GEARS) [3].

Qualitative assessment methods such as OSATS and GEARS are subjective, being based on questionnaires [4,5]. In addition, these methods have limitations in that surgeons need to see and evaluate long-term surgical procedures. An automatic and quantitative method of evaluation for robotic surgery is needed therefore to overcome the limitations of these subjective methods [6]. The main items of OSATS and GEARS are related to the movement of surgical instruments (SIs), resulting in enhanced situational awareness [7]. Application of an SI tracking algorithm to surgical images may automate the evaluation of long-term surgical processes. This evaluation may be quantified by determining the motions of the SI and calculating nine specifically defined motion metrics related to surgical skills.

Current methods of evaluating surgical skills during robotic surgery include the use of the da Vinci Skills Simulator (dVSS). The simulator presents various tasks to surgeons, such as ring and rail, in virtual robotic surgery environments and evaluates surgeons’ proficiencies based on its inbuilt evaluation criteria [8,9]. However, virtual robotic surgery is a practice environment for novice surgeons, differing greatly from actual surgical environments.

Surgical skills have been determined by quantitatively measuring SI movements in actual surgical environments [10,11]. Although kinematics methods estimating mechanical movements of SIs have been used to calculate the relationship between each joint of these SIs [10,11,12,13], these methods can result in cumulative errors in the calculation of the motions of each joint [7]. Moreover, these methods are inapplicable to most surgical robots, except for some research equipment, because they are prevented from approaching the values of kinematic joints [10,11,14].

Image-based methods can directly recognize the SIs in robotic surgery views. Moreover, image-based methods have other advantages because they do not require external equipment and can therefore be applied to surgical robots made by other manufacturers and to laparoscopic surgery. Traditional image processing approaches, however, are limited in detecting SI tips in complex robotic surgery views [15,16]. A deep learning-based approach has been found to overcome these limitations and has been applied to several tasks during robotic surgery, such as classification [17,18], detection [19,20], segmentation [21], and pose estimation [22,23] of SIs, phase identification [24,25], and action recognition [26]. These methods are limited with respect to determining the trajectory of SIs. Semantic segmentation methods applied to robotic surgery images recognize occluded instruments as a single object when the SI locations are close or overlapping [27,28]. Maintenance of the identity of each SI is critical for accurate determination of SI trajectory [29,30]. The identity of SI is easily changed mainly when the SI goes out of the screen or is close to another SI.

The present study proposes a system that quantitatively assesses the surgical skills of a surgeon during robotic surgery by visual tracking of SIs using a deep learning method. The algorithm consists of two frameworks: instance segmentation for occlusion and tracking for maintaining types of SIs. This method was able to stably track the tip positions of SIs in patients with thyroid cancer undergoing robotic thyroid surgery with a bilateral axillo-breast approach (BABA) and in a BABA training model [31,32]. The trajectory of the instruments enabled calculation of defined motion metrics [33], which were used to develop a system for quantitative assessment of surgical skills.

## 2. Materials and Methods

### 2.1. Study Design

**Figure 1:**
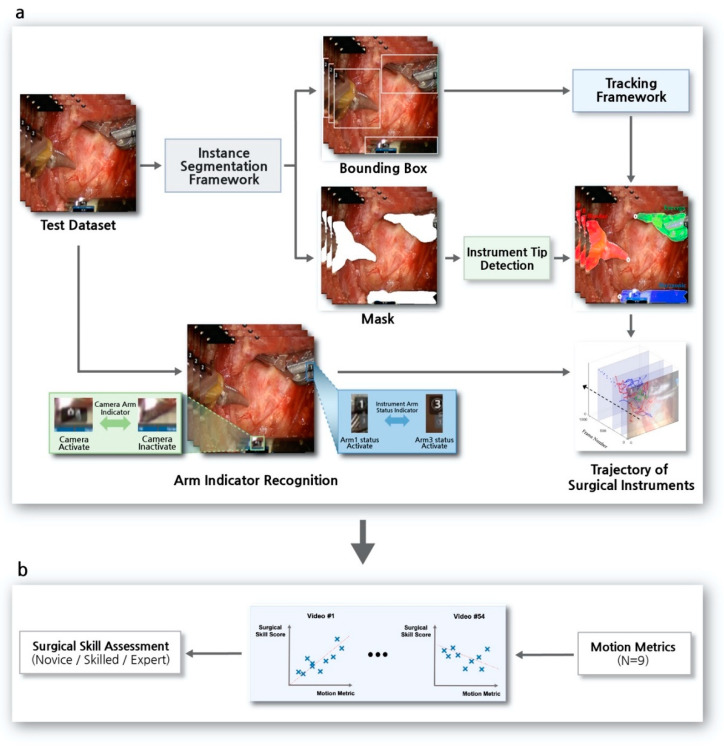
Overview of the surgical skill assessment system in robotic surgery. (**a**) Surgical instrument tracking algorithm. The pipeline consisted of a deep learning-based instance segmentation framework and a tracking framework. Accurate trajectory of the surgical instruments was determined by surgical instrument tip detection and arm-indicator recognition. (**b**) Assessment of surgical skills. Motion metrics (e.g., instruments out of view) were calculated based on the acquired trajectory of surgical instruments and used to develop a surgical skill assessment system.

A deep learning-based tracking algorithm of multiple SIs was developed to assess surgical skills in robotic surgery. Figure 1 shows an overview of the surgical skill assessment system used in robotic surgery. The system consists of two processes, the SI tracking algorithm and the surgical skill assessment.

**Figure 2:**
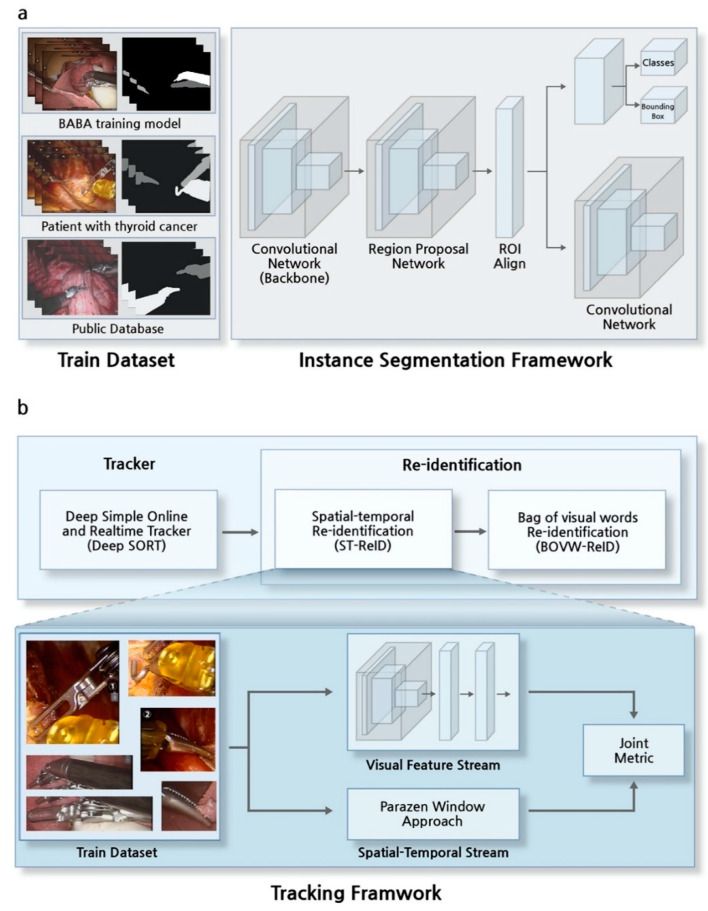
Overview of the instance segmentation and tracking frameworks. (**a**) The instance segmentation framework was trained with three types of training datasets: the bilateral axillo-breast approach (BABA) training model, patients, and a public database. (**b**) The tracking framework, consisting of a tracker and a sequence of re-identification algorithms. Spatial-temporal re-identification (ST-ReID) was trained with bounding boxes of all types of surgical instruments. Bag of visual words re-identification (BOVW-ReID) was applied after ST-ReID.

The SI tracking algorithm is a pipeline of deep learning-based algorithms involving an instance segmentation framework and a tracking framework, along with image processing methods to detect the tips of SIs and to recognize indicators (Figure 1a). The outputs of the instance segmentation framework were a bounding box and a mask of instruments on a surgical view (Figure 2a). The results of the bounding box were input into the tracking framework, involving each SI frame by frame to maintain the type of instruments over time (Figure 2b). The mask results were used to detect the positions of SI tips. To accurately determine the trajectory of each SI, it was necessary to detect the position of its tip, not its center [34]. An indicator recognition algorithm was applied to determine the moment of a laparoscopy usage and the status of an identified SI during robotic surgery. This prevented changes in laparoscopic views and errors due to immobile but present SIs in these views from being included in the trajectory.

Throughout the process of SI tracking, surgical skills were evaluated based on the acquired trajectory. Motion metrics [33] are quantitative indices, mainly related to the movement of SIs [7] in robotic surgical environments (Figure 1b). Nine types of motion metrics were defined, with the metrics calculated based on SI trajectories. In addition, surgical skill scores were determined by surgeons based on selected items related to SI motions in OSATS [2] and GEARS [3]. Finally, calculated motion metrics were used to develop a model predicting the surgical skills of novice, skilled, and expert robotic surgeons. This retrospective study was approved by the Institutional Review Boards of Seoul National University Hospital (IRB No. H-1912-081-1088).

### 2.2. Surgical Procedure

The BABA to robotic thyroid surgery is a minimally invasive method used worldwide [32,35,36]. First, small incisions about 1 cm in size were placed on both sides of the axillae and the breast areolae, and the robot was docked to remove the thyroid gland. A view similar to that of traditional open thyroidectomy and the sophisticated arm movements of the robot provide surgical stability. A BABA training model enabling surgeons to practice has been developed [31]. The video datasets used are segments from the beginning to the locating of the recurrent laryngeal nerve (RLN) during thyroid surgery. Because injury to the RLN is a major complication of thyroid surgery, it is important to preserve RLN function during thyroid surgery [37].

### 2.3. Dataset

Several types of daVinci surgical robots were used (S, Si, and Xi), along with four types of SIs: bipolar, forceps, harmonic, and cautery hook. The developed algorithm was applied to two types of surgical image. The first was a surgical image of a BABA training model developed for thyroid surgery training [31,32]; subjects tested on this image included students, residents, and fellows. The second was a surgical image of a patient with thyroid cancer; subjects tested on this image included fellows and professors.

These two surgical images were used as training and test datasets. Training datasets were used for two kinds of deep learning-based frameworks. The dataset used to train the instance segmentation framework consisted of 84 frames from the BABA training model, 454 frames from patients, and 1766 frames from an open database [38]. The data used to train spatial-temporal re-identification (ST-ReID) in the tracking framework consisted of 253 frames from patients (Table 1).

Test datasets consisted of 14 videos from the BABA training model and 40 videos from patients. Test video lengths ranged from 1121 to 40,621 frames, with a 23 fps. A detailed description of the test datasets is given in Supplementary Table S1.

### 2.4. Instance Segmentation Framework

The instance segmentation framework, Mask R-CNN [39], consisted of sequence algorithms of region proposal networks (RPN) [41] for detecting SIs and semantic segmentation networks (Figure 2a). Unlike semantic segmentation methods applied to robotic surgery images [42,43], the proposed instance segmentation method separates occluded instruments during the first stage of RPN, followed by application of a semantic segmentation network during the next stage. Surgical instruments that were only partially visible on the screen were defined as indistinguishable. Therefore, the datasets were trained using a binary cross-entropy loss to approach a binary rather than a multi-class classification task (Supplementary Figure S1).

### 2.5. Tracker in Tracking Framework

The positions of the SIs determined by the instance segmentation framework, the bounding boxes, must be assigned to the next frame of the same SIs. The tracking framework was designed to associate the identification of an SI to the next identification of that SI and maintain these associations over frames. The framework consisted of a cascade structure, a tracker, and a re-identification method.

The tracker used in this study was a deep simple online and realtime tracker (deep SORT) [26], which associated target SIs in consecutive video frames using spatial and temporal information (Figure 2b). The algorithm operated in the following order. The final bounding box was selected from among the bounding box candidates through a non-maximum suppression method [31] as a result of the instance segmentation framework. Next, the Kalman filter [44] using time information and the intersection-over-union (IOU) using spatial information were applied to associate the identity of SIs that move over time. A Hungarian algorithm was used for optimization of the final selection in association with SIs [45].

### 2.6. Re-Identification in Tracking Framework

Re-identification (ReID) was applied to the result of Deep SORT because the existing identity of an SI can change when an SI moves out of view or when SIs cross in close proximity. In addition, the maximum number of SIs that appear on the robot surgery view was set at three, thus limiting the number of SIs.

In the proposed ReID method, offline and online learning algorithms were applied sequentially. ST-ReID [40] is an offline learning algorithm that trains all types of SIs in advance, whereas bag of visual words re-identification (BOVW-ReID) [46] is an online learning method applied for secondary verification. The moment the prior ST-ReID predicted changes in the identity of SIs, the visual features of the SIs during 10 previous frames were trained, and BOVW-ReID was applied.

### 2.7. Arm-Indicator Recognition on the Robotic Surgery View

The arm indicators that could have affected the trajectory consisted of instrument arm status and camera arm indicators. The instrument arm status indicator on both sides of the screen indicated the SI currently in use. Therefore, these indicators reflected the movement of two or fewer SIs actually being used rather than the movement of the SI that appeared in the robotic surgery view. Recognition of the camera arm indicator confirmed the movement of the laparoscope during the operation. The appearance of the camera arm indicator on the robotic surgery view indicated movement of the laparoscope; however, movement of the screen may have incorrectly indicated movement of the SI. Although varying according to the type of surgical robot, the positions of both indicators were fixed on the view and appeared when an event occurred. To recognize the arm-indicator, template matching [47] was applied to the robotic surgery view. Because the shape and the position of the indicators were fixed, the template of each arm-indicator was stored in advance.

### 2.8. Surgical Skill Prediction Model using Motion Metrics

Two surgeons reviewed recorded videos of surgeons being trained using the BABA training model and of surgeons performing thyroid surgery on patients with thyroid cancer [48,49]. Parts of items and related motion metrics in OSATS and GEARS were scored [31,50]. The defined items included time and motion, instrument handling, and flow of operation and forward planning in OSATS, as well as bimanual dexterity, efficiency, and robotic control in GEARS. Each item was scored from one to five with a total of 15 grades, as shown in Supplementary Table S2.

Based on the acquired trajectories, motion metrics, mainly related movements of SIs, were used to develop a surgical skill prediction model. Seven metrics associated with motion were included [9,33,51]: time to completion of surgery, instruments out of view, instrument collision, economy of motion, average speed, number of movements, and economic factors. Two additional metrics related to the robotic surgery environment, surgical instrument changes and laparoscopy usage, were also included (Supplementary Table S3).

The surgical skill prediction models were developed using these nine calculated motion metrics as well as ground truth from OSATS and GEARS scores. The total number of tested videos was 54, with these datasets divided into 40 training and 12 test sets. Surgical skill prediction models were developed using machine learning methods, a linear classifier, a support vector machine (SVM), and random forest, with the model predicting three groups consisting of novice, skilled, and expert surgeons.

In the training process, five-fold cross validation was applied, and the class imbalance issue was solved by applying the synthetic minority over-sampling technique (SMOTE) [52]. SVM used the Gaussian kernel, with the external hyperparameter optimized through training being a regularization parameter. Additionally, the random forest was trained based on the Gini impurity, with the external hyperparameters optimized through training being the number of trees and the maximum depth of the tree. The selected hyperparameters were trained and fine-tuned during 500 epochs.

## 3. Results

### 3.1. Results of the Instance Segmentation Framework

**Figure 3:**
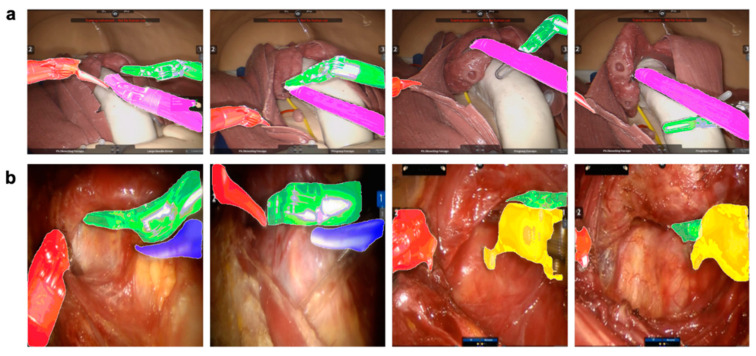
Qualitative results of the instance segmentation framework. Recognition of occlusion between surgical instruments located close together or overlapping (red: bipolar (**i**); pink: bipolar (**ii**); green: forceps; blue: harmonic; yellow: cautery hook). (**a**) Application of sample results to the bilateral axillo-breast approach (BABA) training model. (**b**) Application of sample results to patients.

Figure 3 shows the qualitative results of the instance segmentation framework in the BABA training model and in a patient with thyroid cancer. This result shows that, even when occlusion occurs between surgical instruments, each instrument can be recognized. Supplementary Video S1 also shows the segmentation results for the videos.

### 3.2. Evaluation of the Tracking Framework

Cumulative matching characteristics (CMC) [53], shown in Equation (1), were used to evaluate the proposed tracking method at the moment the identity of SI predicted by the previous deep SORT algorithm was not maintained. Table 2 shows the comparative performance of ReID methods. Before applying the ReID methods, when only Deep SORT was applied, the accuracy of applying the ReID method was measured by setting the ratio of the identity of SIs to 0% as a reference point. The evaluation metric ranked at most three types of SI samples according to their distances to the query. The combination of ST-ReID with BOVW-ReID showed accuracy 93.3% with the BABA training model and 88.1% in patients with thyroid cancer.

(1) $$Accuracy_{1}=\{\begin{array}{c} 1\\ 0 \end{array}\begin{array}{c} \;\;\;\;\;\;if\;top1\;ranked\;SI\;samples\;contatin\;the\;query\;identity\\ otherwise \end{array}$$

### 3.3. Trajectory of Multiple Surgical Instruments and Evaluation

**Figure 4:**
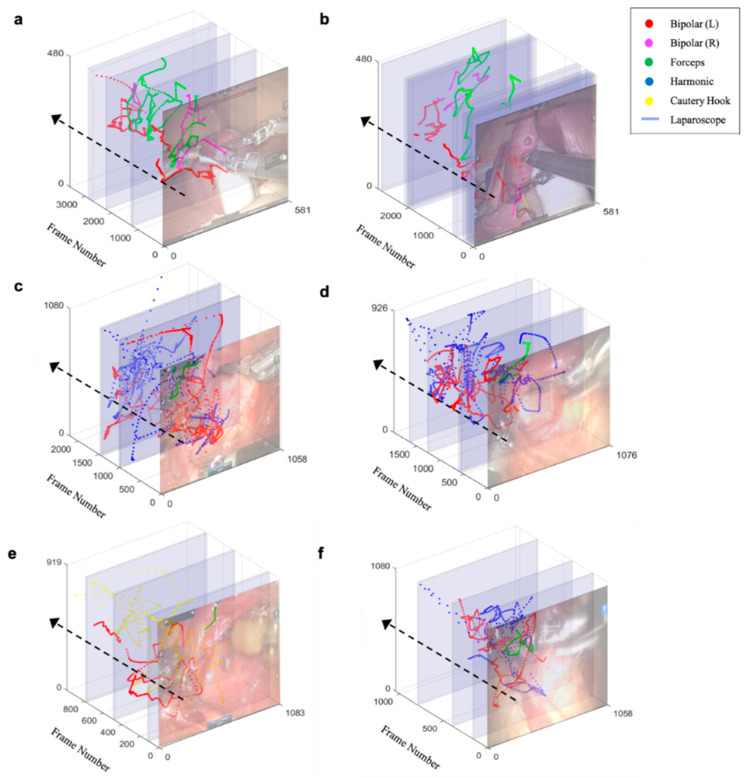
Trajectory of multi-surgical instrument tip. Each color represents a type of surgical instrument, and the blue area represents the duration of laparoscopy. (**a**,**b**) Trajectory of novice surgeons. (**c**,**d**) Trajectory of skilled surgeons. (**e**,**f**) Trajectory of expert surgeons.

Figure 4 shows the trajectory of multi-SI tip, as determined by the proposed tracking algorithm. Procedure for detecting the SI tip is described in Supplementary Materials and Supplementary Figure S2. The differences between the algorithm-based determination of the tip position and the ground truth, labeled at 2 frames per second (23 frames), were determined. The root mean squared error (RMSE) averaged 2.83 mm for the BABA training model and 3.75 mm in patients with thyroid cancer.

The unit of distance that each SI moved was converted from pixels to millimeters because the width and the height of each image were dependent on the type of da Vinci robot used. Thus, depending on the degree of magnification of the laparoscope, errors may have occurred when calculating the movement of the actual SIs. For unit conversion, the thickness of the surgical instrument was measured in advance (8 mm), and the thickness shown on the first screen was measured in pixels units. Therefore, through proportional relationships, the motion of each SI in pixels was converted to millimeters in all surgical images [54].

This system also measured whether the end position of the SI predicted by the algorithm was within 1, 2, and 5 mm of the end position of the SI on the screen [22]. True positive and false positive results were obtained using a confusion matrix. Therefore, area under the curve (AUC) could be calculated by plotting a receiver operating characteristic (ROC) curve using true positive and false positive rates (Supplementary Figure S3).

The mean AUC for errors within 1, 2, and 5 mm were 0.73, 0.83, and 0.92, respectively, in the BABA training model and 0.69, 0.76, and 0.84, respectively, in patients with thyroid cancer. Finally, Pearson’s correlation analysis, performed to assess the similarity between predicted trajectories and ground truth, showed that these trajectories were 0.93 (*x*-axis) and 0.91 (*y*-axis) in the BABA training model and 0.89 (*x*-axis) and 0.86 (*y*-axis) in thyroid cancer patients (Table 3).

### 3.4. Performance of the Surgical Skill Prediction Model using Motion Metrics

The OSATS and the GEARS scores of the two surgeons showed intra-class correlation coefficients (ICC) of 0.711 and 0.74, respectively. Each motion metric item was normalized to the operation time and then to the metric.

**Figure 5:**
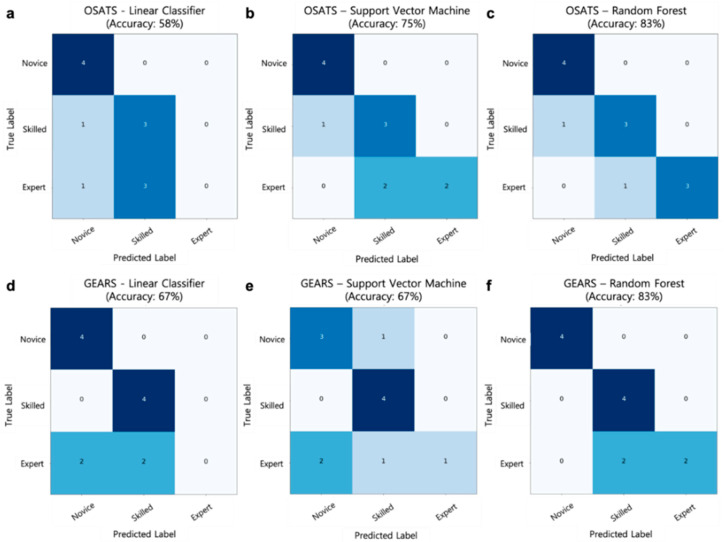
Comparison of the performance of surgical skill prediction models and parts of items in Object Structured Assessment of Technical Skills (OSATS) and Global Evaluative Assessment of Robotic Surgery (GEARS) with a confusion matrix. The test dataset consisted of four novice, four skilled, and four expert surgeons. (**a**–**c**) Confusion matrix results of models using the OSATS. (**a**) Linear classifier; (**b**) support vector machine; and (**c**) random forest. (**d**–**f**) Confusion matrix results of models using the GEARS. (**d**) Linear classifier; (**e**) support vector machine; and (**f**) random forest.

**Figure 6:**
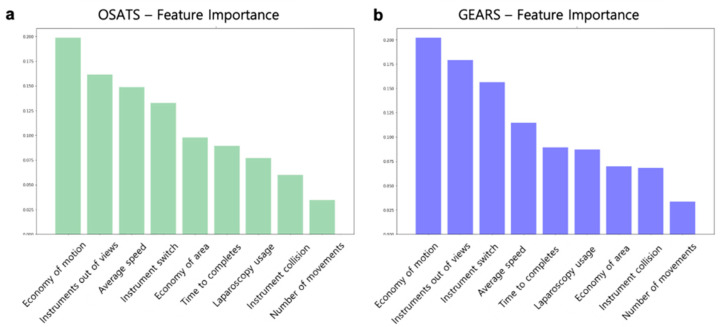
Relative importance of motion metrics in surgical skill prediction models. (**a**) Importance of motion metrics in Object Structured Assessment of Technical Skills (OSATS). (**b**) Importance of motion metrics in Global Evaluative Assessment of Robotic Surgery (GEARS).

The performances of a linear classifier, SVM, and random forest surgical skill prediction models were compared. The models were optimized by hyperparameter tuning, with the random forest showing the highest accuracy. The random forest model had the highest performance and accuracy of 83% with OSATS and 83% with GEARS. Figure 5 shows a comparison of the performance of these surgical skill prediction models. In addition, the relative importance of motion metrics was analyzed in OSATS and GEARS. As shown in Figure 6, the most important metric in both OSATS and GEARS was economy of motion, followed by the instrument being out of view.

## 4. Discussion

To the best of our knowledge, this is the first deep learning-based visual tracking algorithm developed for a quantitative surgical skill assessment system. Conventional methods of evaluating surgical skills such as OSATS [2] and GEARS [3] were based on assessments of recorded videos during robotic surgery from 0 to 30 grades. Because SI movements are associated with surgical skills, the newly proposed quantitative assessment method used a tracking algorithm to determine the trajectories of multiple SIs, showing an accuracy of 83% when compared with conventional methods.

Previously described SI tracking algorithms are limited by occlusion among different SIs and by multiple SIs being recognized as a single SI [27,28]. SI identity cannot be maintained over time because SIs have similar appearances, especially when only parts are visible [55,56]. The proposed method overcomes occlusion using an instance segmentation framework and overcomes identity maintenance using a tracking framework. Accurate determination of SI trajectories enables the calculation of motion metrics and the quantitative evaluation of surgical skills.

The SI tracking algorithm was developed based on robotic surgical environments. In this study, four types of SIs were used, but if only the shaft of the SI appeared on the surgical screen, it could not be discerned, thus we approached the binary classification problem that distinguishes the SI (foreground) from the background. In addition, SI may be difficult to discern when it is covered by tissues or when only a part is visible during surgery [20]. Therefore, to minimize errors resulting from segmentation, the tracking algorithm used temporal information to determine the type of SI. The described tracking algorithm is typically used in a tracking framework to track pedestrians [30,40]. The maximum number of SIs viewed during robotic surgery is three, limiting the number of objects recognized by the proposed algorithms. An arm-indicator recognition algorithm was applied to reflect a robotic surgery environment in which an SI appears but does not actually move. Specifically, the instrument arm status indicator provides information about the two activated SIs in use, with the camera arm indicator determining the moment the laparoscope was moved, preventing errors resulting from the trajectory of the immobile SI.

Our findings also confirmed that the four most important metrics in OSATS and GEARS were the same: economy of motion, instruments out of view, average speed, and instrument switch. The video datasets used in this study were video segments from the beginning of surgery to the locating of the RLN during thyroid surgery. Therefore, the relative importance of the motion metrics may differ depending on surgical sites and tasks.

This study had several limitations. First, the proposed system was applied to video sets of training model and patients with thyroid cancer who underwent BABA surgery. It is necessary to verify the effectiveness of the proposed system using various surgical methods and surgical areas.

Second, we could not directly compare the performances of the kinematics and proposed image-based methods because access to the da Vinci Research Interface is limited, allowing most researchers only to obtain kinematic raw data [10]. However, previous studies have reported that the kinematics method using da Vinci robot had an error of at least 4 mm [57]. Direct comparison of performance is difficult because the surgical images used in the previous study and in this study differed. However, the average RMSE of the proposed image-based tracking algorithm was 3.52 mm, indicating that this method is more accurate than the kinematics method and that the latter cannot be described as superior.

The performance of the current method with the previous visual method could not be directly compared because no similar study detected and tracked the tip coordinates of the SIs. However, studies have used deep learning-based detection methods to determine the bounding boxes of the SIs and to display the trajectory of the center points of these boxes [19,20]. Nevertheless, because this approach could not determine the specific locations of the SIs, it cannot be considered an accurate tracking method intuitively. Comparison of the quantitative performance of the proposed method and other approaches is important, making it necessary to compare different SI tracking methods.

Third, because SIs are detected on two-dimensional views, errors may occur due to the absence of depth information. Errors of magnification were therefore minimized by measuring the width of SIs on the view and converting pixels to millimeters. However, methods are needed to utilize three-dimensional information based on stereoscopic matching of left and right images during robotic surgery [58,59].

Fourth, because the proposed method is a combination of several algorithms, longer videos can result in the accumulation of additional errors, degrading the performance of the system. Thus, in particular, it is necessary to train additional negative examples with the instance segmentation framework, which is the beginning of the pipeline. For example, gauze or tubes on the robotic surgery view can be recognized as SIs (Supplementary Figure S4).

Finally, because errors from re-identification in the tracking framework could critically affect the ability to determine correct trajectories, accurate assessment of surgical skills requires manual correction of errors (Supplementary Figure S5).

## 5. Conclusions

The system proposed in this study can track surgical SIs automatically using deep learning-based visual tracking methods and enable the quantitative assessment of surgical skills. This proposed system may effectively educate students who require training in robotic surgery and will improve the surgical skills of surgeons accustomed to performing robotic surgery.

## Figures and Tables

**Table 1:** Training datasets for the instance segmentation framework and spatial-temporal re-identification.

Training Dataset	No. of Videos	Total No. of Frames	Types of Surgical Instrument			
			Bipolar	Forceps	Harmonic	Cautery Hook
BABA training model (Instance Segmentation Framework [39])	10	84	158	82	-	-
Patients with thyroid cancer (Instance Segmentation Framework [39])	2	454	311	194	141	311
Public database [38] (Instance Segmentation Framework [39])	8	1766	1.451	1351	-	-
Patients with thyroid cancer (ST-ReID [40])	3	253	99	77	81	58

BABA, bilateral axillo-breast approach; ST-ReID, spatial-temporal re-identification.

**Table 2:** Comparative performance of re-identification methods.

ReID Method	BABA Training Model (Rank-1)	Patients with Thyroid Cancer (Rank-1)
BOVW-ReID [46]	68.3%	57.9%
ST-ReID [40]	91.7%	85.2%
BOVW-ReID [46] + ST-ReID [40]	93.3%	88.1%

ReID, Re-identification; BABA, bilateral axillo-breast approach; BOVW-ReID, bag of visual words re-identification; ST-ReID, spatial-temporal re-identification.

**Table 3:** Comparative performance of methods detecting the tips of surgical instruments. Evaluation methods included determinations of average root mean square error (RMSE; mm), average area under the curve (AUC; 1, 2 and 5 mm), and average Pearson correlation coefficient (*x*-axis and *y*-axis) between tip positions determined by the algorithm and ground truth.

Test Dataset (No. of Videos)	No. of Frames	RMSE (mm)	AUC (1 mm)	AUC (2 mm)	AUC (5 mm)	Pearson-r (*x*-axis)	Pearson-r (*y*-axis)
BABA training model (*n* = 14)	125,984	2.83 ± 1.34	0.73 ± 0.05	0.83 ± 0.02	0.92 ± 0.02	0.93 ± 0.02	0.91 ± 0.04
Patients with thyroid cancer (*n* = 40)	387,884	3.7 ± 2.29	0.69 ± 0.04	0.76 ± 0.06	0.84 ± 0.03	0.89 ± 0.03	0.86 ± 0.03
Average (*n* = 54)	513,868	3.52 ± 2.12	0.7 ± 0.05	0.78 ± 0.06	0.86 ± 0.05	0.9 ± 0.03	0.87 ± 0.04

BABA, bilateral axillo-breast approach; RMSE, root mean square error; AUC, area under the curve; Pearson-r, Pearson’s correlation coefficient.

## Supplementary Materials

The following are available online at https://www.mdpi.com/2077-0383/9/6/1964/s1, Video S1: Qualitative result of instance segmentation framework. Figure S1: Plot of model loss on the training and validation datasets. Figure S2: Procedure for detecting the tip of surgical instruments. Figure S3: Area under the curve (AUC) calculation method of the coordinates of the surgical instrument tip predicted by the algorithm. Figure S4: Errors in instance segmentation framework. Figure S5: Errors in tracking framework. Table S1: Description of test datasets. Table S2: Description of Object Structured Assessment of Technical Skills (OSATS) and Global Evaluative Assessment of Robotic Surgery (GEARS) with relevance to motion metrics. Table S3: Description of motion metrics. Motion metrics were defined in reference to the robotic surgical environment, and consist primarily of movements of surgical instruments and numbers of laparoscopes.

## References

[1] Pernar L.I., Robertson F.C., Tavakkoli A., Sheu E.G., Brooks D.C., Smink D.S. (2017). An appraisal of the learning curve in robotic general surgery. Surg. Endosc..

[2] Martin J., Regehr G., Reznick R., Macrae H., Murnaghan J., Hutchison C., Brown M. (1997). Objective structured assessment of technical skill (OSATS) for surgical residents. Br. J. Surg..

[3] Goh A.C., Goldfarb D.W., Sander J.C., Miles B.J., Dunkin B.J. (2012). Global evaluative assessment of robotic skills: Validation of a clinical assessment tool to measure robotic surgical skills. J. Urol..

[4] Takeshita N., Phee S.J., Chiu P.W., Ho K.Y. (2018). Global Evaluative Assessment of Robotic Skills in Endoscopy (GEARS-E): Objective assessment tool for master and slave transluminal endoscopic robot. Endosc. Int. Open.

[5] Hilal Z., Kumpernatz A.K., Rezniczek G.A., Cetin C., Tempfer-Bentz E.-K., Tempfer C.B. (2017). A randomized comparison of video demonstration versus hands-on training of medical students for vacuum delivery using Objective Structured Assessment of Technical Skills (OSATS). Medicine.

[6] Ponto J. (2015). Understanding and evaluating survey research. J. Adv. Pract. Oncol..

[7] Reiter A., Allen P.K., Zhao T. Articulated surgical tool detection using virtually-rendered templates. Proceedings of the Computer Assisted Radiology and Surgery (CARS).

[8] Mark J.R., Kelly D.C., Trabulsi E.J., Shenot P.J., Lallas C.D. (2014). The effects of fatigue on robotic surgical skill training in Urology residents. J. Robot. Surg..

[9] Brinkman W.M., Luursema J.-M., Kengen B., Schout B.M., Witjes J.A., Bekkers R.L. (2013). da Vinci skills simulator for assessing learning curve and criterion-based training of robotic basic skills. Urology.

[10] Lin H.C., Shafran I., Yuh D., Hager G.D. (2006). Towards automatic skill evaluation: Detection and segmentation of robot-assisted surgical motions. Comput. Aided Surg..

[11] Kumar R., Jog A., Vagvolgyi B., Nguyen H., Hager G., Chen C.C.G., Yuh D. (2012). Objective measures for longitudinal assessment of robotic surgery training. J. Thorac. Cardiovasc. Surg..

[12] Fawaz H.I., Forestier G., Weber J., Idoumghar L., Muller P.-A. (2018). Evaluating surgical skills from kinematic data using convolutional neural networks. arXiv.

[13] Hung A.J., Oh P.J., Chen J., Ghodoussipour S., Lane C., Jarc A., Gill I.S. (2019). Experts vs super-experts: Differences in automated performance metrics and clinical outcomes for robot-assisted radical prostatectomy. BJU Int..

[14] Jun S.-K., Narayanan M.S., Agarwal P., Eddib A., Singhal P., Garimella S., Krovi V. Robotic minimally invasive surgical skill assessment based on automated video-analysis motion studies. Proceedings of the 2012 4th IEEE RAS & EMBS International Conference on Biomedical Robotics and Biomechatronics (BioRob).

[15] Speidel S., Delles M., Gutt C., Dillmann R. Tracking of instruments in minimally invasive surgery for surgical skill analysis. Proceedings of the International Workshop on Medical Imaging and Virtual Reality.

[16] Ryu J., Choi J., Kim H.C. (2013). Endoscopic vision-based tracking of multiple surgical instruments during robot-assisted surgery. Artif. Organs.

[17] Mishra K., Sathish R., Sheet D. Learning latent temporal connectionism of deep residual visual abstractions for identifying surgical tools in laparoscopy procedures. Proceedings of the IEEE Conference on Computer Vision and Pattern Recognition Workshops.

[18] Sahu M., Mukhopadhyay A., Szengel A., Zachow S. (2017). Addressing multi-label imbalance problem of surgical tool detection using CNN. Int. J. Comput. Assist. Radiol. Surg..

[19] Sarikaya D., Corso J.J., Guru K.A. (2017). Detection and localization of robotic tools in robot-assisted surgery videos using deep neural networks for region proposal and detection. IEEE Trans. Med. Imaging.

[20] Choi B., Jo K., Choi S., Choi J. Surgical-tools detection based on Convolutional Neural Network in laparoscopic robot-assisted surgery. Proceedings of the 2017 39th Annual International Conference of the IEEE Engineering in Medicine and Biology Society (EMBC).

[21] García-Peraza-Herrera L.C., Li W., Gruijthuijsen C., Devreker A., Attilakos G., Deprest J., Vander Poorten E., Stoyanov D., Vercauteren T., Ourselin S. Real-time segmentation of non-rigid surgical tools based on deep learning and tracking. Proceedings of the International Workshop on Computer-Assisted and Robotic Endoscopy.

[22] Law H., Ghani K., Deng J. Surgeon technical skill assessment using computer vision based analysis. Proceedings of the Machine Learning for Healthcare Conference.

[23] Kurmann T., Neila P.M., Du X., Fua P., Stoyanov D., Wolf S., Sznitman R. Simultaneous recognition and pose estimation of instruments in minimally invasive surgery. Proceedings of the International Conference on Medical Image Computing and Computer-Assisted Intervention.

[24] Twinanda A.P., Shehata S., Mutter D., Marescaux J., De Mathelin M., Padoy N. (2017). Endonet: A deep architecture for recognition tasks on laparoscopic videos. IEEE Trans. Med. Imaging.

[25] Yu F., Croso G.S., Kim T.S., Song Z., Parker F., Hager G.D., Reiter A., Vedula S.S., Ali H., Sikder S. (2019). Assessment of automated identification of phases in videos of cataract surgery using machine learning and deep learning techniques. JAMA Netw. Open.

[26] Khalid S., Goldenberg M., Grantcharov T., Taati B., Rudzicz F. (2020). Evaluation of deep learning models for identifying surgical actions and measuring performance. JAMA Netw. Open.

[27] García-Peraza-Herrera L.C., Li W., Fidon L., Gruijthuijsen C., Devreker A., Attilakos G., Deprest J., Vander Poorten E., Stoyanov D., Vercauteren T. ToolNet: Holistically-nested real-time segmentation of robotic surgical tools. Proceedings of the 2017 IEEE/RSJ International Conference on Intelligent Robots and Systems (IROS).

[28] Pakhomov D., Premachandran V., Allan M., Azizian M., Navab N. (2017). Deep residual learning for instrument segmentation in robotic surgery. arXiv.

[29] Zheng L., Shen L., Tian L., Wang S., Wang J., Tian Q. Scalable person re-identification: A benchmark. Proceedings of the IEEE International Conference on Computer Vision.

[30] Wojke N., Bewley A., Paulus D. Simple online and realtime tracking with a deep association metric. Proceedings of the 2017 IEEE International Conference on Image Processing (ICIP).

[31] Yu H.W., Yi J.W., Seong C.Y., Kim J.-k., Bae I.E., Kwon H., Chai Y.J., Kim S.-j., Choi J.Y., Lee K.E. (2018). Development of a surgical training model for bilateral axillo-breast approach robotic thyroidectomy. Surg. Endosc..

[32] Lee K.E., Kim E., Koo D.H., Choi J.Y., Kim K.H., Youn Y.-K. (2013). Robotic thyroidectomy by bilateral axillo-breast approach: Review of 1026 cases and surgical completeness. Surg. Endosc..

[33] Oropesa I., Sánchez-González P., Chmarra M.K., Lamata P., Fernández A., Sánchez-Margallo J.A., Jansen F.W., Dankelman J., Sánchez-Margallo F.M., Gómez E.J. (2013). EVA: Laparoscopic instrument tracking based on endoscopic video analysis for psychomotor skills assessment. Surg. Endosc..

[34] Jin A., Yeung S., Jopling J., Krause J., Azagury D., Milstein A., Fei-Fei L. (2018). Tool detection and operative skill assessment in surgical videos using region-based convolutional neural networks. arXiv.

[35] Liu S.Y.-W., Kim J.S. (2017). Bilateral axillo-breast approach robotic thyroidectomy: Review of evidences. Gland Surg..

[36] He Q., Zhu J., Zhuang D., Fan Z., Zheng L., Zhou P., Yu F., Wang G., Ni G., Dong X. (2019). Robotic lateral cervical lymph node dissection via bilateral axillo-breast approach for papillary thyroid carcinoma: A single-center experience of 260 cases. J. Robot. Surg..

[37] Christou N., Mathonnet M. (2013). Complications after total thyroidectomy. J. Visc. Surg..

[38] Allan M., Shvets A., Kurmann T., Zhang Z., Duggal R., Su Y.-H., Rieke N., Laina I., Kalavakonda N., Bodenstedt S. (2019). 2017 robotic instrument segmentation challenge. arXiv.

[39] He K., Gkioxari G., Dollár P., Girshick R. Mask r-cnn. Proceedings of the 2017 IEEE International Conference on Computer Vision (ICCV).

[40] Wang G., Lai J., Huang P., Xie X. Spatial-temporal person re-identification. Proceedings of the AAAI Conference on Artificial Intelligence.

[41] Girshick R. Fast r-cnn. Proceedings of the IEEE International Conference on Computer Vision.

[42] Laina I., Rieke N., Rupprecht C., Vizcaíno J.P., Eslami A., Tombari F., Navab N. Concurrent segmentation and localization for tracking of surgical instruments. Proceedings of the International Conference on Medical Image Computing and Computer-Assisted Intervention.

[43] Shvets A., Rakhlin A., Kalinin A.A., Iglovikov V. (2018). Automatic instrument segmentation in robot-assisted surgery using deep learning. arXiv.

[44] Bishop G., Welch G. (2001). An introduction to the kalman filter. Proc SIGGRAPH Course.

[45] Kuhn H.W. (1955). The Hungarian method for the assignment problem. Nav. Res. Logist. Q..

[46] Peng X., Wang L., Wang X., Qiao Y. (2016). Bag of visual words and fusion methods for action recognition: Comprehensive study and good practice. Comput. Vis. Image Underst..

[47] Yoo J.-C., Han T.H. (2009). Fast normalized cross-correlation. Circuits Syst. Signal Process..

[48] Yu C., Yang S., Kim W., Jung J., Chung K.-Y., Lee S.W., Oh B. (2018). Acral melanoma detection using a convolutional neural network for dermoscopy images. PLoS ONE.

[49] Yamazaki Y., Kanaji S., Matsuda T., Oshikiri T., Nakamura T., Suzuki S., Hiasa Y., Otake Y., Sato Y., Kakeji Y. (2020). Automated surgical instrument detection from laparoscopic gastrectomy video images using an open source convolutional neural network platform. J. Am. Coll. Surg..

[50] Vernez S.L., Huynh V., Osann K., Okhunov Z., Landman J., Clayman R.V. (2017). C-SATS: Assessing surgical skills among urology residency applicants. J. Endourol..

[51] Pagador J.B., Sánchez-Margallo F.M., Sánchez-Peralta L.F., Sánchez-Margallo J.A., Moyano-Cuevas J.L., Enciso-Sanz S., Usón-Gargallo J., Moreno J. (2012). Decomposition and analysis of laparoscopic suturing task using tool-motion analysis (TMA): Improving the objective assessment. Int. J. Comput. Assist. Radiol. Surg..

[52] Chawla N.V., Bowyer K.W., Hall L.O., Kegelmeyer W.P. (2002). SMOTE: Synthetic minority over-sampling technique. J. Artif. Intell. Res..

[53] Paisitkriangkrai S., Shen C., Van Den Hengel A. Learning to rank in person re-identification with metric ensembles. Proceedings of the IEEE Conference on Computer Vision and Pattern Recognition.

[54] Lee D., Yu H.W., Kim S., Yoon J., Lee K., Chai Y.J., Choi J.Y., Kong H.-J., Lee K.E., Cho H.S. (2020). Vision-based tracking system for augmented reality to localize recurrent laryngeal nerve during robotic thyroid surgery. Sci. Rep..

[55] Reiter A., Allen P.K., Zhao T. (2014). Appearance learning for 3D tracking of robotic surgical tools. Int. J. Robot. Res..

[56] Reiter A., Allen P.K., Zhao T. Learning features on robotic surgical tools. Proceedings of the 2012 IEEE Computer Society Conference on Computer Vision and Pattern Recognition Workshops (CVPRW).

[57] Nisky I., Hsieh M.H., Okamura A.M. The effect of a robot-assisted surgical system on the kinematics of user movements. Proceedings of the 2013 35th Annual International Conference of the IEEE Engineering in Medicine and Biology Society (EMBC).

[58] Allan M., Ourselin S., Thompson S., Hawkes D.J., Kelly J., Stoyanov D. (2013). Toward detection and localization of instruments in minimally invasive surgery. IEEE Trans. Biomed. Eng..

[59] Allan M., Chang P.-L., Ourselin S., Hawkes D.J., Sridhar A., Kelly J., Stoyanov D. Image based surgical instrument pose estimation with multi-class labelling and optical flow. Proceedings of the International Conference on Medical Image Computing and Computer-Assisted Intervention.

