# Supplementary material for: Evaluation of Surgical Skills during Robotic Surgery by Deep Learning-Based Multiple Surgical Instrument Tracking in Training and Actual Operations
Source: J Clin Med. 2020 Jun 23;9(6):1964. doi: 10.3390/jcm9061964 (PMC7355689; doi:10.3390/jcm9061964)
Supplement: Supplementary file 1 [file jcm-09-01964-s001.zip › Supplementary_final.docx]

Supplementary Materials

**Video S1.** Qualitative result of instance segmentation framework.

Surgical instrument tip detection

The SI tip detection algorithm was applied because the tip position more accurately reflected the movement of the SI than a detection algorithm tracking the center of each SI bounding box [1]. SI tips were detected by applying a binary SI mask, which resulted from the instance segmentation framework. The starting point was determined by considering the number of SI masks contacted among eight defined sections a certain distance from the edge of the view. A skeletonization algorithm was applied to the SI mask [2], and the position of the SI tip in the skeletonized SI was determined by calculating the longest accumulated distance from the starting point. Finally, the kalman filter was applied to minimize outliers [3,4] (Figure S2).


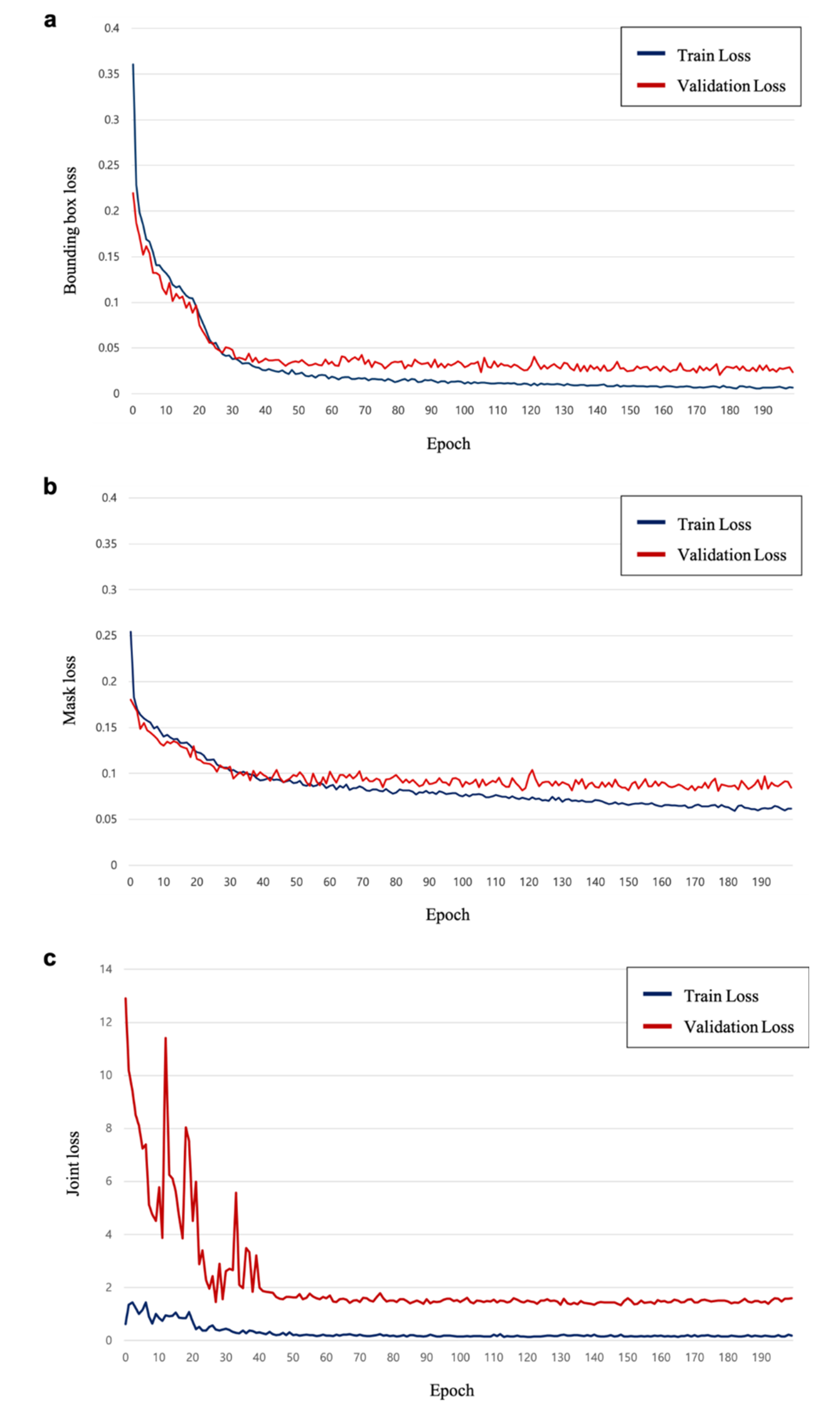


Figure S1. Plot of model loss on the training and validation datasets. (a) Loss of bounding box in the instance segmentation framework. (b) Loss of mask in the instance segmentation framework. (c) Loss of spatial-temporal re-identification.


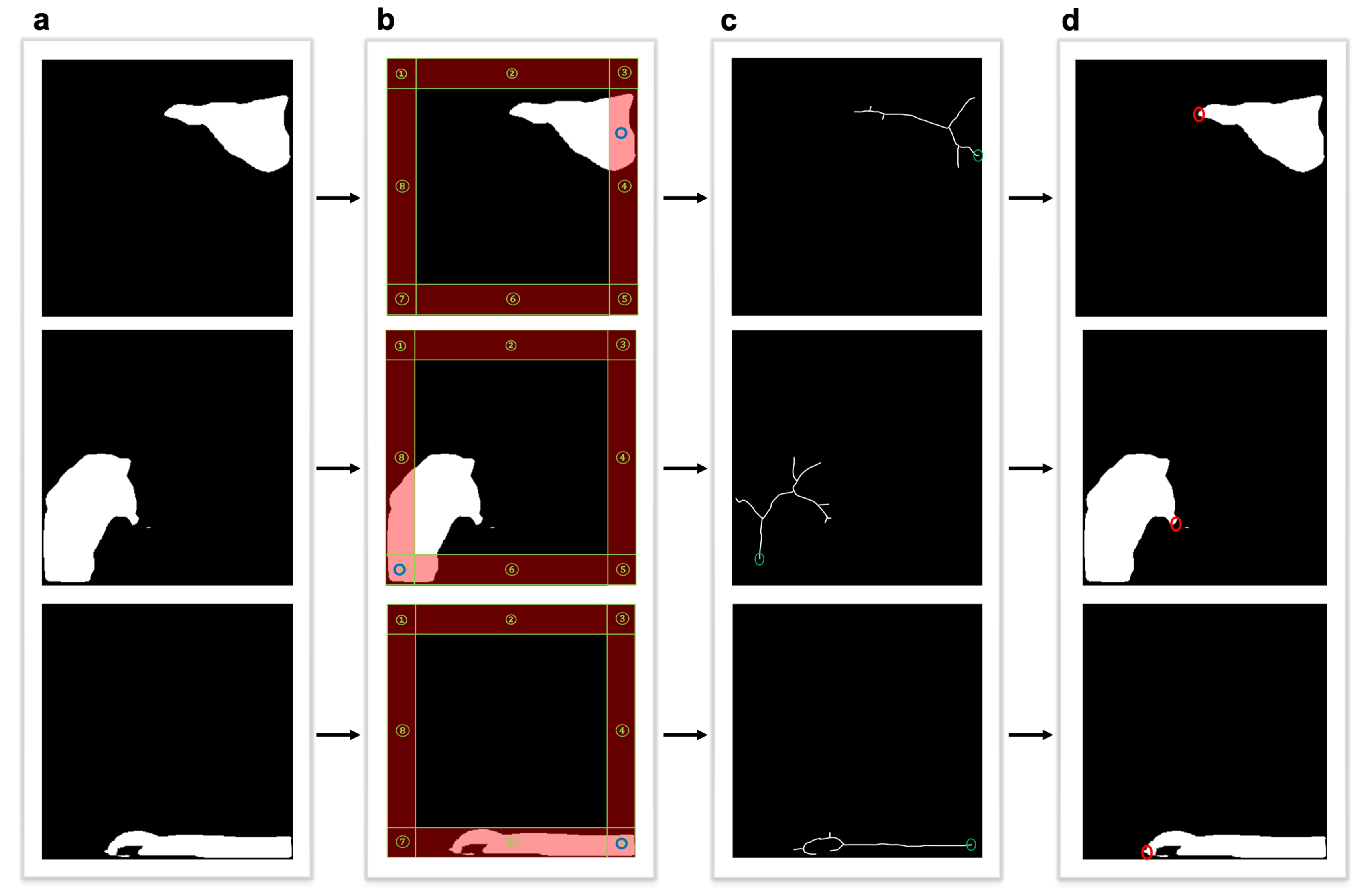


**Figure S2.** Procedure for detecting the tip of surgical instruments. (a) Surgical instrument mask from the instance segmentation framework. (b) Starting point detection in the mask from edges of the view. The area located at a certain distance from the edge, and the area at which the mask overlaps was determined. The starting point (blue) was based on the number of contacted sections at a certain distance from the edge of the view. (c) Application of the skeletonization algorithm to the mask to determine the main skeleton. After calculating the skeleton, update the position of the starting point (green) to the nearest position in the skeleton. (d) Determination of the tool tip position (red) within the skeleton by calculating the longest accumulated distance from the starting point, and kalman filter was applied.


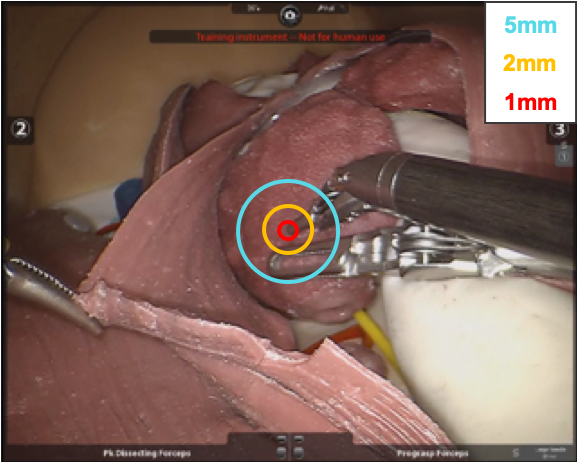


Figure S3. Area under the curve (AUC) calculation method of the coordinates of the surgical instrument tip predicted by the algorithm. Red, yellow, and blue circles indicate the range of 1, 2, and 5mm from the surgical instrument tip, respectively.


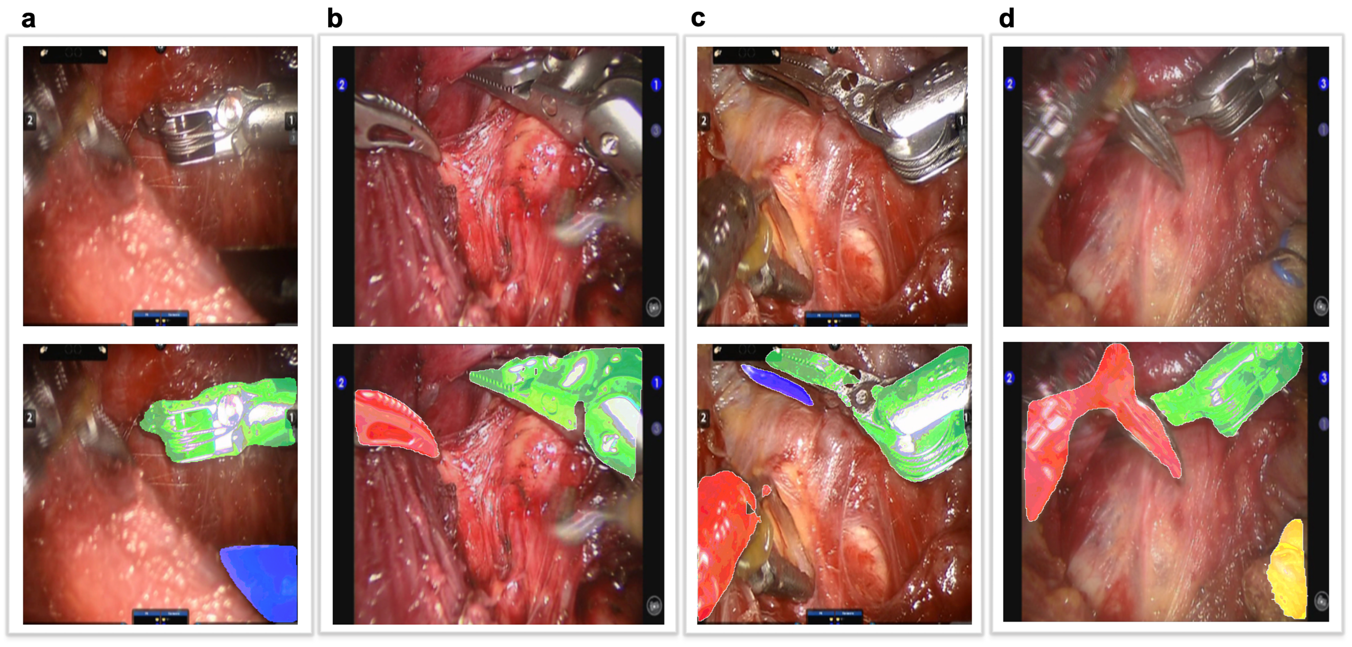


**Figure S4.** Errors in instance segmentation framework. (Top: Original, Bottom: Result of instance segmentation framework) (a-b). False negatives resulting from sudden movements of a surgical instrument. ©. False positive, in which part of a surgical instrument is recognized as a single object. (d). False positive case, in which a non-surgical instrument is recognized as a surgical instrument.


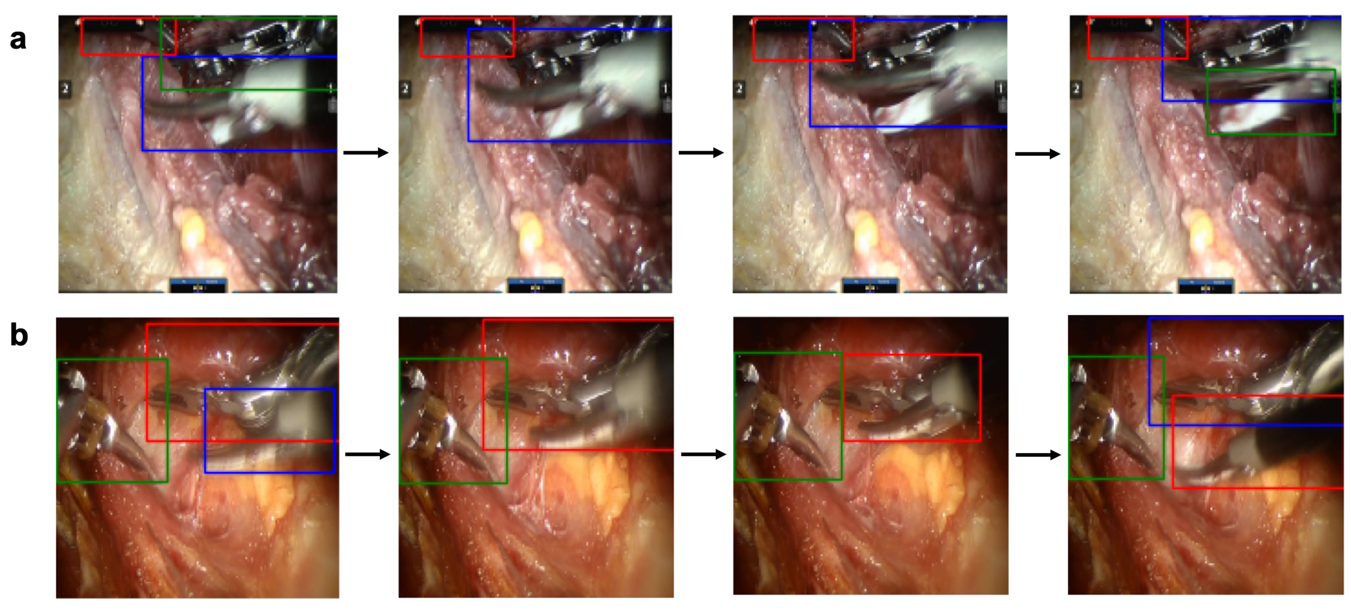


Figure S5. Errors in tracking framework. (a-b). Errors in which the identity of surgical instruments was switched following occlusion.

**Table S1.** Description of test datasets. The test dataset consisted of 14 videos of the axillo-breast approach (BABA) to thyroid surgery and 40 videos of patients. The number of scenes is the number of segmented videos at the point of time when laparoscopy was used.

| **Test dataset** | **Video no.** | **No. of scenes** | **Total no. of frames** | **Video no.** | **No. of scenes** | **Total no. of frames** |
| --- | --- | --- | --- | --- | --- | --- |
| **BABA training model** | 1 | 6 | 11,087 | 8 | 1 | 7,639 |
|  | 2 | 7 | 6,978 | 9 | 6 | 10,687 |
|  | 3 | 11 | 7,638 | 10 | 3 | 10,744 |
|  | 4 | 4 | 10,086 | 11 | 1 | 10,763 |
|  | 5 | 6 | 7,323 | 12 | 1 | 7,298 |
|  | 6 | 2 | 6,140 | 13 | 2 | 11,174 |
|  | 7 | 2 | 11,285 | 14 | 2 | 7,142 |
|  | Total | 54 | 125,984 | **-** | **-** | **-** |
| **Patients with thyroid cancer** | 1 | 2 | 2,869 | 21 | 19 | 22,748 |
|  | 2 | 6 | 16,489 | 22 | 5 | 3,570 |
|  | 3 | 2 | 7,262 | 23 | 4 | 8,375 |
|  | 4 | 8 | 32,846 | 24 | 16 | 2,832 |
|  | 5 | 3 | 6,260 | 25 | 8 | 11,227 |
|  | 6 | 8 | 15,401 | 26 | 5 | 8,483 |
|  | 7 | 6 | 18,587 | 27 | 5 | 1,875 |
|  | 8 | 7 | 6,909 | 28 | 11 | 9,212 |
|  | 9 | 7 | 18,249 | 29 | 10 | 13,359 |
|  | 10 | 5 | 11,967 | 30 | 21 | 40,621 |
|  | 11 | 2 | 8,202 | 31 | 2 | 2,831 |
|  | 12 | 3 | 4,710 | 32 | 4 | 5,587 |
|  | 13 | 5 | 8,407 | 33 | 5 | 3,452 |
|  | 14 | 1 | 2,357 | 34 | 3 | 3,511 |
|  | 15 | 8 | 9,787 | 35 | 16 | 7,140 |
|  | 16 | 7 | 11,046 | 36 | 22 | 20,494 |
|  | 17 | 6 | 6,009 | 37 | 9 | 9,209 |
|  | 18 | 2 | 1,121 | 38 | 13 | 3,584 |
|  | 19 | 5 | 11,236 | 39 | 4 | 3,307 |
|  | 20 | 5 | 4,613 | 40 | 1 | 2,140 |
|  | Total | 281 | 387,884 | - | - | - |

**Table S2.** Description of Object Structured Assessment of Technical Skills (OSATS) and Global Evaluative Assessment of Robotic Surgery (GEARS) with relevance to motion metrics.

| **No.** | **Object Structured Assessment of**  **Technical Skills [9]** | **Relevance to motion metrics** | **Global Evaluative Assessment**  **of Robotic Surgery [10]** | **Relevance to motion metrics** |
| --- | --- | --- | --- | --- |
| 1 | Respect for tissue | X | Depth of perception | X |
| 2 | Time and motion | O | Bimanual dexterity | O |
| 3 | Instrument handling | O | Efficiency | O |
| 4 | Knowledge of instruments | X | Force sensitivity | X |
| 5 | Use of assistants | X | Autonomy | X |
| 6 | Flow of operation and  forward planning | O | Robotic control | O |
| 7 | Knowledge of  specific procedure | X | - | - |

**Table S3.** Description of motion metrics. Motion metrics were defined in reference to the robotic surgical environment, and consist primarily of movements of surgical instruments and numbers of laparoscopes.

| **No.** | **Motion metrics** | **Description** |
| --- | --- | --- |
| 1 | Time to complete (s) [11] | Total time from the beginning to end of all surgical procedures |
| 2 | Instruments out of views (s) [11] | Total distance traveled by all instruments when not in view |
| 3 | Instrument collision (n) [11] | Number of times one instrument collided with another instrument |
| 4 | Economy of motion (mm) [11] | Total distance traveled by instruments |
| 5 | Average speed (mm/s) [12] | Rate of change of the instrument’s position in the image |
| 6 | Number of movement (n) [13] | Number of times beyond an acceleration of tolerance threshold |
| 7 | Economy of Area (-) [12] | Relationship between the maximum image area occupied by the instrument and the total distance |
| 8 | Instrument switch (n) | Number of surgical instrument type changes |
| 9 | Laparoscopy usage (n) | Number of times of laparoscopy usage |

**References**

1. Jin, A.; Yeung, S.; Jopling, J.; Krause, J.; Azagury, D.; Milstein, A.; Fei-Fei, L. Tool Detection and Operative Skill Assessment in Surgical Videos Using Region-Based Convolutional Neural Networks. *arXiv preprint arXiv:1802.08774* **2018**.

2. Saha, P.K.; Borgefors, G.; di Baja, G.S. A survey on skeletonization algorithms and their applications. *Pattern Recognition Letters* **2016**, *76*, 3-12.

3. Bishop, G.; Welch, G.; Greg. *An Introduction to the Kalman Filter*. Proc of SIGGRAPH, Course, **2001**, *8*, 41.

4. Ryu, J.; Choi, J.; Kim, H.C. Endoscopic vision‐based tracking of multiple surgical instruments during robot‐assisted surgery. *Artificial organs* **2013**, *37*, 107-112.

5. Yoo, J.-C.; Han, T.H. Fast normalized cross-correlation. *Circuits, systems and signal processing* **2009**, *28*, 819.

6. He, K.; Gkioxari, G.; Dollár, P.; Girshick, R. Mask r-cnn. In Proceedings of Computer Vision (ICCV), 2017 IEEE International Conference on; pp. 2980-2988.

7. Allan, M.; Shvets, A.; Kurmann, T.; Zhang, Z.; Duggal, R.; Su, Y.-H.; Rieke, N.; Laina, I.; Kalavakonda, N.; Bodenstedt, S. 2017 Robotic instrument segmentation challenge. *arXiv preprint arXiv:1902.06426* **2019**.

8. Wang, G.; Lai, J.; Huang, P.; Xie, X. Spatial-temporal person re-identification. In Proceedings of Proceedings of the AAAI Conference on Artificial Intelligence; pp. 8933-8940.

9. Martin, J.; Regehr, G.; Reznick, R.; Macrae, H.; Murnaghan, J.; Hutchison, C.; Brown, M. Objective structured assessment of technical skill (OSATS) for surgical residents. *British journal of surgery* **1997**, *84*, 273-278.

10. Goh, A.C.; Goldfarb, D.W.; Sander, J.C.; Miles, B.J.; Dunkin, B.J. Global evaluative assessment of robotic skills: validation of a clinical assessment tool to measure robotic surgical skills. *The Journal of urology* **2012**, *187*, 247-252.

11. Brinkman, W.M.; Luursema, J.-M.; Kengen, B.; Schout, B.M.; Witjes, J.A.; Bekkers, R.L. da Vinci skills simulator for assessing learning curve and criterion-based training of robotic basic skills. *Urology* **2013**, *81*, 562-566.

12. Oropesa, I.; Sánchez-González, P.; Chmarra, M.K.; Lamata, P.; Fernández, A.; Sánchez-Margallo, J.A.; Jansen, F.W.; Dankelman, J.; Sánchez-Margallo, F.M.; Gómez, E.J. EVA: laparoscopic instrument tracking based on endoscopic video analysis for psychomotor skills assessment. *Surgical endoscopy* **2013**, *27*, 1029-1039.

13. Pagador, J.B.; Sánchez-Margallo, F.M.; Sánchez-Peralta, L.F.; Sánchez-Margallo, J.A.; Moyano-Cuevas, J.L.; Enciso-Sanz, S.; Usón-Gargallo, J.; Moreno, J. Decomposition and analysis of laparoscopic suturing task using tool-motion analysis (TMA): improving the objective assessment. *International journal of computer assisted radiology and surgery* **2012**, *7*, 305-313.
